# Evaluation of the PATHFAST TB LAM Ag assay as a treatment monitoring tool for pulmonary tuberculosis in Nairobi, Kenya

**DOI:** 10.1186/s41182-025-00771-z

**Published:** 2025-07-01

**Authors:** Fred Orina, Mayu Hikone, Nobuo Saito, Jane Ong’ang’o, Andrew Nyerere, Edinah Songoro, Helen Meme

**Affiliations:** 1 Center for Respiratory Disease Research, Kenya Medical Research Institute, Nairobi, Kenya; 2 Kenya Research Station, Institute of Tropical Medicine, Nagasaki University, Nagasaki, Japan; 3 Department of Medical Microbiology, College of Health Sciences, Jomo Kenyatta University of Agriculture and Technology, Nairobi, Kenya

**Keywords:** Lipoarabinomannan, Pulmonary tuberculosis, Biomarker, Treatment monitoring tool

## Abstract

**Background:**

Treatment monitoring is important in pulmonary tuberculosis (PTB) management, since prolonged treatment necessitates regular assessments to prevent treatment failure and the emergence of drug-resistant strains. However, the lack of a simple, rapid, and reliable treatment monitoring tool (TMT) remains a major challenge. We evaluated the utility of measuring sputum lipoarabinomannan (LAM) concentration by the PATHFAST TB LAM Ag assay (PHC Corporation, Tokyo, Japan) as a TMT in patients with PTB in Nairobi, Kenya.

**Methods:**

We retrospectively analyzed sputum LAM levels via the PATHFAST TB LAM Ag assay from a Nairobi cohort of patients with PTB and compared these results with conventional microbiological tests (acid-fast bacilli [AFB] smear microscopy; mycobacterial growth indicator tube [MGIT] culture). Stored sputum pellets processed with N-acetyl-L-cysteine (NALC)-NaOH were used for LAM measurement. Serial LAM concentrations measured every 2 weeks over an 8-week period were compared across bacterial load categories to assess correlations with AFB smear grades and culture results using the Kruskal–Wallis and Mann–Whitney U tests.

**Results:**

The 98 patients included here had a median age of 37 years (Interquartile Range: 27–44). The majority were men (74/98, 75.5%) and the MGIT culture was positive for 89 (90.8%) of them. Patients with elevated baseline LAM concentrations showed a significant reduction in LAM levels with treatment (90% median reduction by week 8), whereas those with low baseline LAM concentrations did not show a declining trend. Sputum LAM levels were significantly higher in culture-positive samples compared to culture-negative samples (23.8 pg/mL vs. 10.8 pg/mL, *P* < 0.001). Sputum LAM levels showed a significant correlation with AFB smear grades, with median concentrations increasing progressively from 11.3 pg/mL in smear-negative samples to 19.7 pg/mL in scanty/1 + samples, and 46.7 pg/mL in 2 + /3 + samples (*P* = 0.0001). LAM levels were significantly higher in culture-positive/AFB-positive sputum samples (viable bacilli) than in culture-negative/AFB-positive samples (non-viable bacilli) (*P* < 0.0001).

**Conclusion:**

Our findings revealed that sputum LAM concentration declined during TB treatment, particularly among patients with high baseline levels, and correlated with AFB smear grades and culture results. Additionally, LAM concentrations differed between culture-positive and culture-negative samples among AFB smear-positive samples. Further prospective studies are needed to assess LAM levels as a TMT.

## Introduction

Treatment failure in tuberculosis (TB) poses a significant challenge to global TB control efforts, accelerating worse clinical outcomes and a worldwide spread of disease, while driving the development of drug-resistant TB [1]. Effective treatment monitoring tools (TMTs) are essential for early identification of treatment failure, enabling timely interventions to control disease progression and the spread of drug-resistant TB. However, the lack of simple and rapid TMTs remains a major challenge in clinical practice [2].

Currently, the standard methods for monitoring treatment response, assessing disease progression, and detecting treatment failure in pulmonary TB (PTB) include clinical assessment, microbiological tests, and radiological evaluation. Microbiological tests, such as acid-fast bacilli (AFB) smear microscopy and culture, are used to quantify the bacterial load in sputum and assess treatment response. Although commonly used, these conventional methods have several limitations [3]. AFB smear microscopy is quick and available in primary care settings; however, its sensitivity is limited and unable to differentiate viable from non-viable bacilli [4]. Although mycobacterial culture is considered the gold standard for assessing bacterial load and treatment efficacy, it has limited availability in most healthcare facilities in low- and middle-income countries exhibiting a high TB burden due to high costs, the need for high-level biosafety laboratory infrastructure, and delayed turnaround times to provide a timely feedback [5]. Given the existing limitations and challenges, there is a growing need for a simple and rapid method for effective TB treatment monitoring [6, 7]. To facilitate the development of such TMTs, evaluation recommendations have been published [6].

Lipoarabinomannan (LAM), a component of the *Mycobacterium tuberculosis* (MTB) cell wall, is one of the pathogen-based biomarkers of MTB that has been extensively studied as a diagnostic marker, particularly as a urinary antigen [8]. However, current lateral flow assay kits for urinary LAM have not been established for treatment monitoring. Efforts have been made to utilize serial measurements of sputum LAM concentrations as a TMT [9–16]. A study developed an enzyme-linked immunosorbent assay (ELISA)-based method to measure sputum LAM concentration, detecting all smear- and culture-positive samples (n = 70) and 50% (n = 29) of smear-negative but culture-positive samples (n = 58), while showing no positivity in non-TB cases (n = 56). Additionally, LAM concentrations inversely correlated with mycobacterial growth indicator tube (MGIT) time-to-detection (TTD) (Pearson correlation, *r* = − 0.803), indicating that sputum LAM concentration reflects bacterial burden in patients with PTB and may serve as a biomarker of treatment response [16]. While the LAM-ELISA test provides results faster than culture methods (within 5 h), it still faces challenges in achieving shorter measurement times, a wider dynamic range, simpler operation, and reliable correlation with bacterial load during treatment. To enhance TB treatment monitoring, the PATHFAST TB LAM Ag assay (PHC Corporation, Tokyo, Japan) was developed as a simple, automated chemiluminescent enzyme immunoassay (CLEIA) that quantifies LAM in sputum within 1 h, including manual sample pretreatment. This cartridge-based assay, utilizing CLEIA and MAGTRATION technology, offers a ready-to-use solution for rapid and standardized LAM quantification [10]. A study has evaluated the analytical performance of the PATHFAST TB LAM Ag assay using biobank samples to measure sputum LAM concentration. The assay demonstrated a sensitivity of 88.8% and a specificity of 100% for detecting culture-positive TB at a 10 pg/mL cutoff, making it comparable to the Xpert MTB/RIF. Additionally, LAM concentration correlated strongly with MGIT TTD (Spearman’s rank correlation, *r* = − 0.770) [10]. While the PATHFAST TB LAM Ag assay has shown potential as a rapid and reliable tool for measuring LAM using biobank samples, evidence supporting its longitudinal performance during TB treatment remains limited. Existing data have primarily focused on analytical validation, with little implications for high TB burden, resource-limited settings such as sub-Saharan Africa.

This study aimed to describe longitudinal trends in sputum LAM levels during TB treatment and to characterize the relationship between sputum LAM concentration and conventional monitoring tools (i.e., AFB smear microscopy, culture) among patients with PTB in Nairobi, Kenya. Additionally, we sought to examine the potential clinical utility of quantifying sputum LAM concentration as an effective TMT.

## Methods

### Study design and setting

This study was nested within a larger cohort study that recruited 362 patients with PTB to investigate the genetic diversity of MTB isolates from bacteriologically confirmed patients with PTB. Among the 326 participants enrolled in the larger cohort, the first 300 participants were included in a follow-up scheme in which every third participant (n = 100) was followed every 2 weeks until two consecutive sputum samples tested negative by AFB smear microscopy. Although microscopy results were used to determine the end of follow-up, sputum samples collected at each visit were also subjected to culture and LAM testing as part of the sub-study. The current sub-study described here utilized serial sputum samples from these followed up participants to assess the relationship between sputum LAM levels and conventional TMTs (i.e., AFB smear microscopy, culture).

The main cohort study recruited participants between June 2023 and August 2024 from the Rhodes Chest Clinic, a public healthcare facility in Nairobi County, Kenya, equipped with a dedicated TB clinic. The inclusion criteria for the main cohort study were newly diagnosed adult patients with PTB (aged ≥ 15 years) who had undergone baseline sputum testing for TB by either the Xpert MTB/RIF Ultra assay (Cepheid, CA, USA) or based on AFB smear positivity. Patients already receiving anti-TB treatment at the time of enrollment were excluded. For this sub-study, among either Xpert assay-positive or AFB-positive patients who were followed up (n = 100), we retrospectively analyzed stored serial sputum samples specifically selecting those who were either Xpert assay-positive or MGIT culture-positive at baseline. As the study aimed to examine the relationship between sputum LAM concentration and bacterial load indicators (AFB smear and culture), inclusion was not restricted by rifampin susceptibility status determined by Xpert assay. Patients who were found to be TB-positive via sputum AFB smear but did not undergo Xpert MTB/RIF Ultra testing and had negative MGIT culture results were excluded from the analysis.

### Ethical considerations

The study protocol, including the follow-up and assessment of LAM levels using the PATHFAST TB LAM Ag assay, was reviewed and approved by the Scientific Ethics Review Unit (SERU) Ethics Committee of the Kenya Medical Research Institute (KEMRI) (Ethical Approval Number: SERU 4595). All participants provided written informed consent prior to enrollment, including consent for serial sputum collection throughout the follow-up period.

### Study procedures and data

Upon enrollment, research staff collected demographic and clinical data, including symptoms, comorbidities, and history of TB. Hospital staff obtained baseline sputum samples and performed either the Xpert MTB/RIF Ultra assay or AFB smear microscopy using fluorescence microscopy as part of routine hospital care. The hospital staff initiated all participants on standard TB treatment with a fixed-dose combination of isoniazid, rifampin, ethambutol, and pyrazinamide. During the weekly visits of the study participants for prescription refills, research staff collected follow-up sputum samples every 2 weeks for research purposes. Research laboratory staff tested baseline and follow-up sputum samples using MGIT culture, AFB smear microscopy, and the PATHFAST TB LAM Ag assay to quantify LAM levels as part of the study protocol. Participants self-reported previous TB history and comorbidities, while healthcare providers offered routine HIV testing to all patients. The hospital assessed treatment outcomes at 6 months following TB treatment initiation, following the guidelines of the National Tuberculosis, Leprosy, and Lung Disease Program of Kenya. The study did not determine causes of death, and we categorized patients who were lost to follow-up or transferred out as having unknown outcomes.

### Laboratory procedures

Sputum samples were processed using the N-acetyl-L-cysteine (NALC)-NaOH method [17]. The resulting pellets were cultured in liquid media using the BACTEC MGIT 960 system (Becton, Dickinson and Company, Sparks, USA) for mycobacterial growth, and AFB smear microscopy was performed using Ziehl–Neelsen staining. Positive cultures were confirmed as MTB using the Capilia TB-Neo assay (Tauns Laboratories, Japan).

### PATHFAST TB LAM Ag assay

LAM extraction and measurement procedures were carried out following the manufacturer’s instructions [10]. While either raw sputum or NALC-NaOH processed sputum sample is acceptable as a primary sample [10, 18], we used NALC-NaOH processed sputum pellets that had been stored at − 30 °C in the KEMRI freezer for durations raging from 1 to 14 months. Briefly, after thawing the sputum pellet at room temperature, 100 µL of 1 N NaOH was added to 200 µL of the sample. The mixture was incubated (100 °C, 20 min), neutralized with 50 µL of 5 M NaH_2_PO_4_ and centrifuged (3000 g, 5 min). The supernatant obtained after centrifugation was collected as the LAM extract for analysis. For LAM measurement, 100 µL of the LAM extract was transferred into a reagent cartridge and loaded into the PATHFAST analyzer. The LAM extraction process was performed manually and took approximately 30 min, followed by automated LAM quantification, which required an additional 17 min for result output.

### Statistical analysis

Baseline characteristics of the study population were summarized using descriptive statistics. Continuous variables were described using median values with interquartile ranges (IQRs). Categorical variables were summarized using frequencies and percentages.

Sputum LAM concentrations were summarized using median values with IQRs at baseline and follow-up time points. Patients were stratified into three baseline LAM groups (high: ≥ 100 pg/mL, mid: 10–99 pg/mL, low: < 10 pg/mL) to explore trends in LAM concentration decline during TB treatment. These thresholds were selected based on the distribution of LAM concentrations in our dataset. The lower cutoff (10 pg/mL) reflects the cutoff value indicated by Akinaga et al. [10], while the upper threshold (100 pg/mL) was selected to identify a subgroup with markedly elevated LAM concentrations. We visualized the trends to illustrate these changes over time. To evaluate the trend of LAM levels after TB treatment initiation, the change rate ((LAM_baseline_ − LAM_weeks_)/LAM_baseline_ × 100 (%)) was described for each follow-up time point (at weeks 2, 4, 6, and 8) for high- and mid-baseline groups. These analyses were restricted to participants who achieved treatment success at 6 months in order to characterize LAM concentration trends in those patients with favorable outcomes.

To assess whether the sputum LAM concentration correlates with MGIT culture positivity and AFB smear grade, LAM concentrations were compared across the categories. Median LAM concentration values between two groups were compared using the Mann–Whitney U test, and for comparisons across multiple groups the Kruskal–Wallis test followed by Dunn’s test was used. To further examine LAM concentration in non-viable bacilli (characterized by culture-negative and AFB-positive samples), bacterial load was stratified based on the combined outcomes of MGIT culture and AFB smear results. Three categories were defined: culture-negative/AFB-negative, culture-negative/AFB-positive, and culture-positive/AFB-positive. The analyses were based on paired data between sputum LAM levels and corresponding MGIT culture or AFB smear results obtained from the same time point. For graphical representation, LAM concentrations were log-transformed using base 10 to visualize the distribution.

All statistical tests were two-sided, and statistical significance was defined as *P* < 0.05 or assessed using 95% Confidence Interval. All statistical analyses were performed using STATA software version 18 (Stata Corporation, USA).

## Results

### Baseline characteristics

**Fig. 1:**
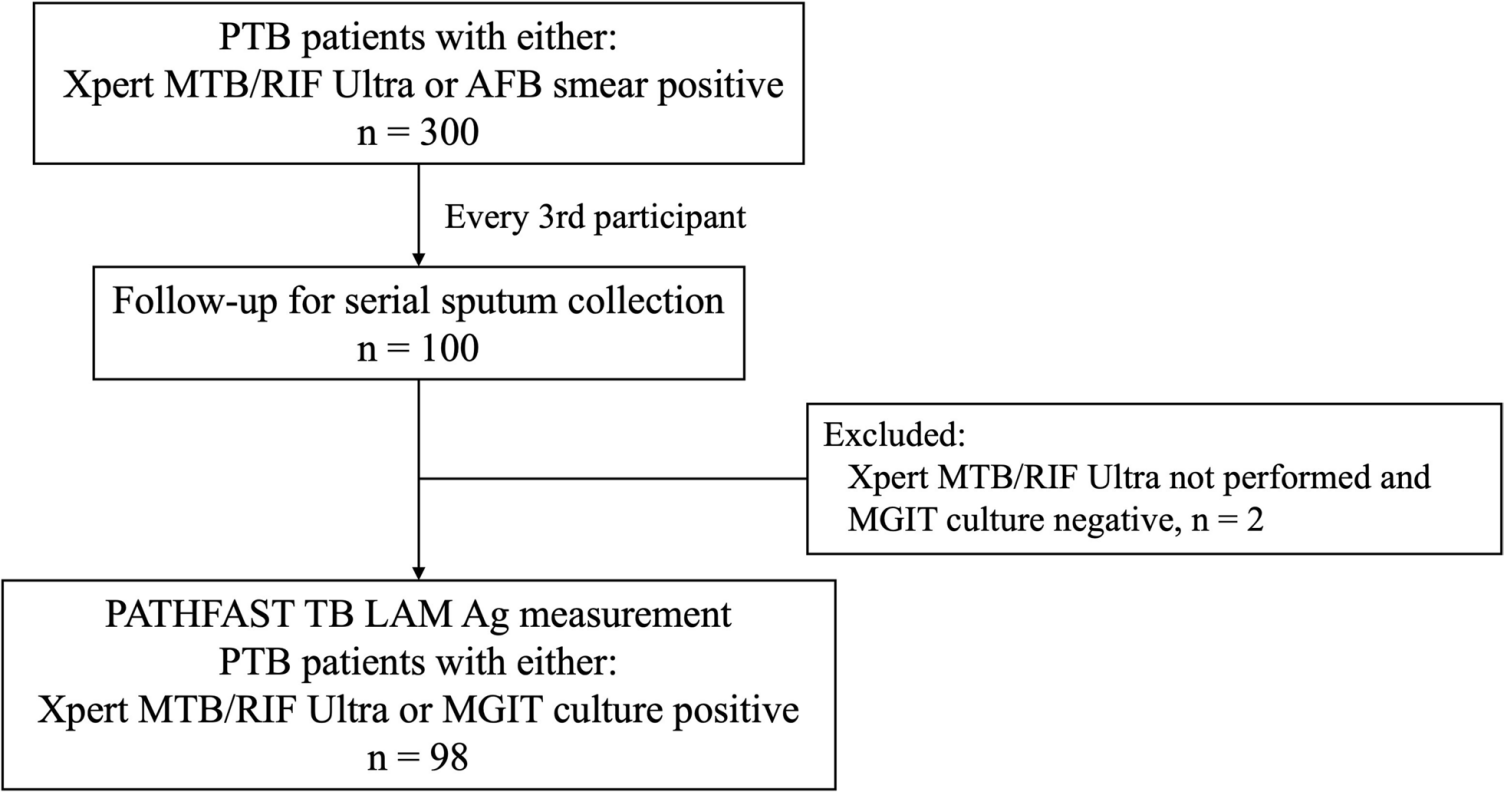
Schematic showing a flow chart of the study. PTB, pulmonary tuberculosis; AFB, acid-fast bacilli; MGIT, mycobacterial growth indicator tube; LAM, lipoarabinomannan

A total of 98 patients were eligible for analysis in this study (Fig. 1). The median duration of follow-up was 6 weeks (IQR: 4–10). The median age of patients was 37 years (IQR: 27–44), predominantly composed of young to middle-aged adults, and the majority were men (n = 74, 75.5%) (Table 1). At the time of enrollment, many patients were found to be underweight and the median body mass index value was 19 kg/m2 (IQR: 18–21). Additionally, 63 patients (64.3%) reported experiencing all four classic TB-related symptoms: cough, fever, night sweats, and weight loss. A previous history of TB was reported by 27 patients (27.6%) and two were HIV-positive (2.0%). At baseline, sputum AFB smear microscopy was performed in 97 patients, with 93 participants (95.9%) testing positive. Xpert MTB/RIF Ultra was positive in 82 participants, among whom three were found to have revealing RIF resistance. Of the 98 patient’s analyzed, most tested positive via MGIT culture (n = 89, 90.8%), with a median TTD value of 9 days (IQR: 6–12). Among these 89 culture-positive patients, 87 were AFB smear-positive, and two were AFB smear-negative. Two patients (2.0%) were negative for both AFB smear and culture testing. Treatment outcomes at 6 months were available for 86 patients, including one reported death and seven classified as treatment failures.

**Table 1:** Baseline characteristics of the study cohort (n = 98)

Variables	N (%)
Age, median [IQR], years	37 [27–44]
Sex, male	74 (75.5)
BMI, median [IQR], kg/m^2^	19 [18–21]
Symptoms	
Cough	98 (100)
Fever	74 (75.5)
Night sweat, n = 97	85 (87.6)
Weight loss	81 (82.7)
Dyspnea	43 (44.9)
Previous history of TB	27 (27.6)
HIV, positive	2 (2.0)
Baseline mycobacterial investigations	
AFB smear result, n = 97	
Negative	4 (4.1)
Scanty	45 (46.4)
1 +	23 (23.7)
2 +	21 (21.7)
3 +	4 (4.1)
Xpert MTB/RIF Ultra result, n = 82	
Trace	5 (6.1)
Low	21 (25.6)
Medium	8 (9.8)
High	48 (58.5)
RIF resistant	3 (3.7)
MGIT culture result	
Positive	89 (90.8)
TTD, median [IQR], days	9 [6–12]
Smear negative and culture negative	2 (2.1)
Smear negative and culture positive	2 (2.1)
Smear positive and culture positive	87 (89.7)
Treatment outcome at 6-months	
Treatment success	78 (79.6)
Treatment failure	7 (7.1)
Death	1 (1.0)
Unknown	12 (12.2)

AFB, acid-fast bacilli; BMI, body mass index; HIV, human immunodeficiency virus; IQR, inter-quartile range; LAM, lipoarabinomannan; MGIT, mycobacterial growth indicator tube; RIF, rifampin; TB, tuberculosis; TTD, time to detection

### Trends of sputum LAM levels during TB treatment

Overall, the median value for baseline sputum LAM concentration was 19.2 pg/mL (IQR: 10.3–56.7). Among the 78 patients who achieved treatment success, baseline LAM concentrations were stratified into three groups: high (≥ 100 pg/mL, n = 14), mid (10–99 pg/mL, n = 43), and low (< 10 pg/mL, n = 18).

**Fig. 2:**
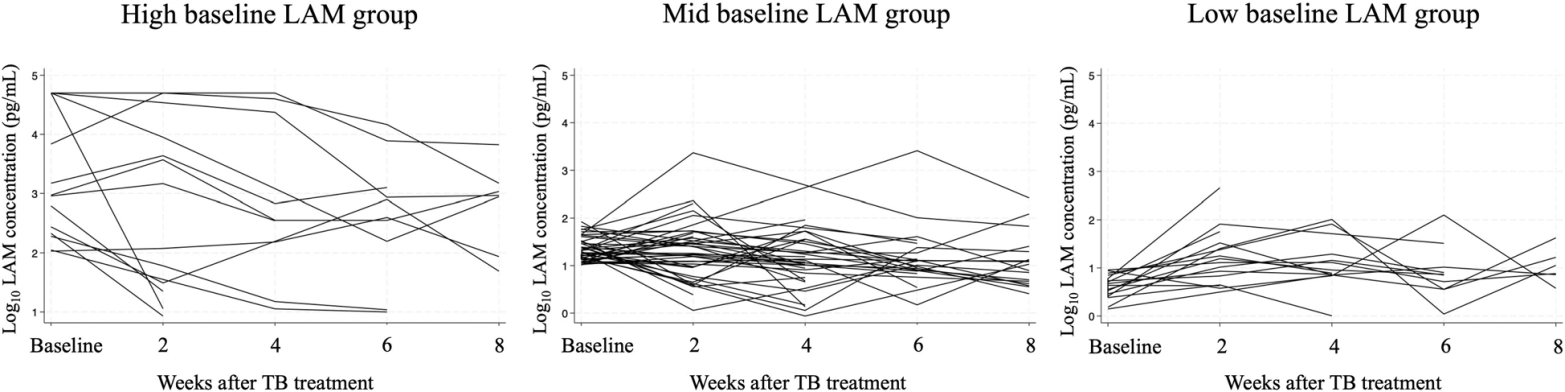
Sputum LAM concentration over time (baseline to week 8 of treatment) for each patient. Footnote: The median change rate of sputum LAM concentration from baseline to week 8 ((LAM_baseline_ − LAM_week8_)/LAM_baseline_ × 100 (%)) for high baseline group (baseline LAM ≥ 100 pg/mL, n = 8), mid baseline group (baseline LAM 10–99 pg/mL, n = 17) and low baseline group (baseline LAM < 10 pg/mL, n = 6) were, − 90% (IQR: − 98 to − 11), − 55% (IQR: − 81–28) and 277% (− 16–639), respectively. IQR, interquartile range; LAM, lipoarabinomannan; TB, tuberculosis

**Fig. 3:**
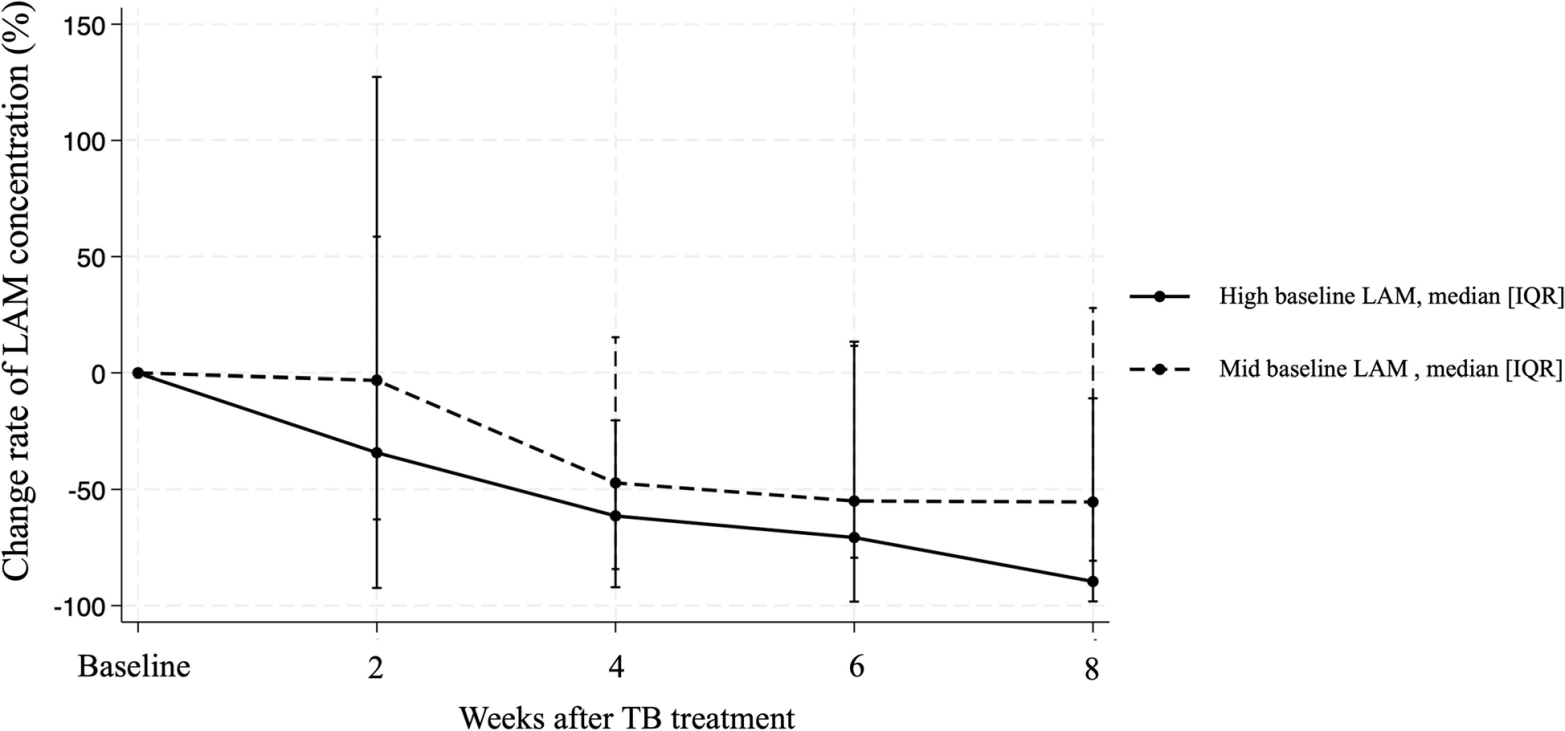
Rate of change of sputum LAM concentration after TB treatment initiation. Footnote: Median change rate of LAM concentration (%) at week 2, 4, 6 and 8 for the high baseline LAM group was − 34% (IQR: − 92–127), − 61% (IQR: − 92 to − 20), − 71% (IQR: − 98–13), and − 90% (IQR: − 98 to − 11), respectively. For the mid baseline LAM group, the corresponding changes were − 3% (IQR: − 63–59), − 47% (IQR: − 84–15), − 55% (IQR: − 79–12), and − 55% (IQR: − 81–28). IQR, interquartile range; LAM, lipoarabinomannan; TB, tuberculosis

The sputum LAM concentration trends over time are presented in Fig. 2 based on baseline LAM levels. Overall, the median change rate for LAM concentration from baseline to week 8 was − 37% (IQR: − 84–42). The median change rate values for LAM concentrations from baseline to week 8 was − 90% (IQR: − 98 to − 11) in the high baseline group, − 55% (IQR: − 81–28) in the mid group, and 277% (− 16–639) in the low group. Figure 3 shows the trend of change rate compared to baseline sputum LAM levels and each follow-up point (week 2, 4, 6 and 8 after TB treatment initiation) for the high- and mid-baseline LAM groups.

### Sputum LAM concentration stratified by culture and AFB smear results

**Fig. 4:**
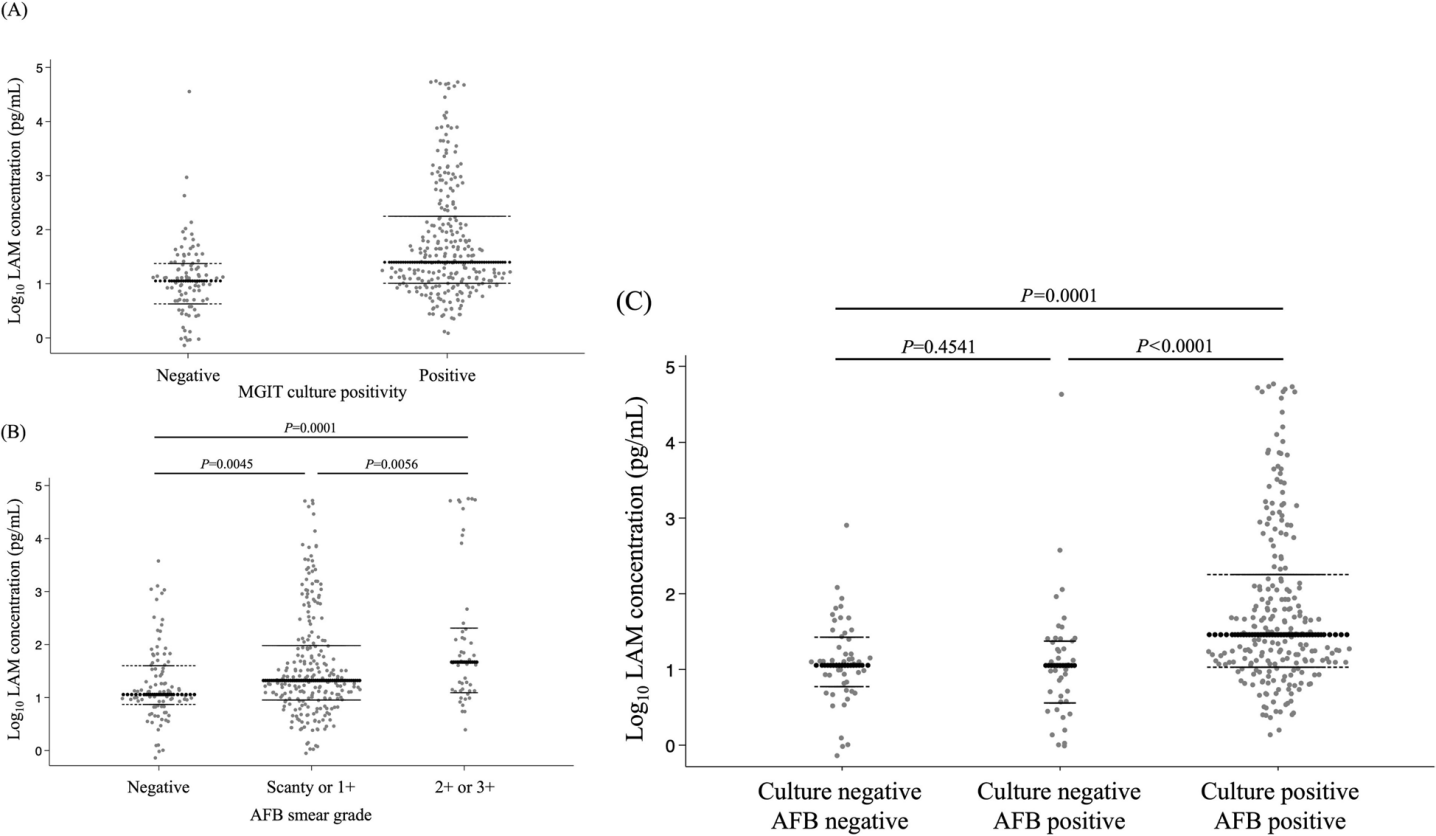
Sputum LAM concentration measurements stratified by culture and AFB smear results. **a** Association between LAM concentration and MGIT culture results (positive vs. negative). **b** LAM concentration according to AFB smear grade. **c** LAM concentration across bacterial load categories. Footnote: **a** Median LAM concentration in MGIT culture-negative (n = 95) and -positive (n = 264) samples were, 10.8 pg/mL (IQR: 4.2–24.8) and 23.8 pg/mL (IQR: 10.4–155.0), respectively (*P* < 0.001). **b** Median LAM concentration in AFB smear grade, negative (n = 97), scanty/1 + (n = 239) and 2 +/3 + (n = 48) were, 11.3 pg/mL (IQR: 6.9–41.4), 19.7 pg/mL (IQR: 8.11–98.1) and 46.7 pg/mL (IQR: 13.1–216.0), respectively (Kruskal–Wallis, *P* = 0.0001). **c** Median LAM concentration in culture-negative/AFB-negative (n = 70), culture-negative/AFB-positive (n = 46), and culture-positive/AFB-positive (n = 248) were, 11.1 pg/mL (IQR: 4.5–24.8), 10.9 pg/mL (IQR: 3.3–25.0) and 25.1 pg/mL (IQR: 11.0–148.0), respectively (Kruskal–Wallis,* P* = 0.0001). AFB, acid-fast bacilli; IQR, interquartile range; LAM, lipoarabinomannan; MGIT, mycobacterial growth indicator tube; TB, tuberculosis

The median LAM concentration value was higher in MGIT culture-positive compared to culture-negative patient groups (23.8 pg/mL [IQR: 10.4–155.0] vs. 10.8 pg/mL [IQR: 4.2–24.8], *P* < 0.001) (n = 362) (Fig. 4a). The median LAM concentration values among smear-negative, scanty/1 + and 2 +/3 + patient groups were, 11.3 pg/mL (IQR: 6.9–41.4), 19.7 pg/mL (IQR: 8.1–98.1) and 46.7 pg/mL (IQR: 13.1–216.0), respectively (n = 384) (Fig. 4b).

The median LAM concentration value did not significantly differ between the culture-negative/AFB-negative and culture-negative/AFB-positive groups (11.1 pg/mL [IQR: 4.5–24.8] vs. 10.9 pg/mL [IQR: 3.3–25.0], *P* = 0.4541). However, a significantly higher median LAM concentration value was observed in the culture-positive/AFB-positive group compared with the culture-negative/AFB-positive group (25.1 pg/mL [IQR: 11.0–148.0] vs. 10.9 pg/mL [IQR: 3.3–25.0], *P* < 0.0001) (Fig. 4c).

## Discussion

In this study, we retrospectively analyzed sputum LAM concentrations using the PATHFAST TB LAM Ag assay in a cohort from Nairobi, Kenya. Notably, the cohort reflected real-world conditions, including variability in sample quality and the use of a NALC-NaOH processed pellet for LAM measurement. Our findings offer valuable insights into the pragmatic utility of the assay in endemic, resource-limited settings.

The description of sputum LAM concentration trends during TB treatment enables assessment of the potential utility of sputum LAM levels as a biomarker for monitoring treatment response. Our findings revealed significant variation in the change rate of LAM levels across a range of different baseline levels. Among the 78 patients who achieved treatment success, the median change rate of LAM concentration from baseline to week 8 of treatment was − 37% (IQR: − 84–42). This overall reduction in LAM concentration aligns with the expected bactericidal effect of TB treatment, with the decline in LAM levels corresponding to a reduction in bacterial load. A similar pattern of LAM concentration reduction has been reported by Kawasaki et al. and Jones et al., where LAM concentrations measured by the LAM-ELISA assay declined in response to effective TB treatment [14, 16]. However, the substantial IQR values ranging from − 84 to 42% highlight the significant variability in LAM concentration dynamics. This variability could be due to differences in factors such as baseline disease severity, immune response, drug susceptibility or treatment adherence. Further investigation is needed to better understand these factors and to assess whether LAM concentration trends could be used to predict individual treatment outcomes.

Our data revealed distinct trends based on baseline LAM concentrations, which were categorized into high (≥ 100 pg/mL), mid (10–99 pg/mL) and low (< 10 pg/mL) LAM concentration groups. Patients with higher baseline LAM levels experienced the most pronounced LAM decline with treatment, indicating strong early bactericidal activity. In the mid-baseline LAM concentration group, the reduction was less pronounced with treatment but was still statistically significant. This group demonstrated a more moderate decrease in LAM concentration, which may imply a slower bacterial clearance process or a less aggressive response to treatment. Individuals in this category may require more careful monitoring to ensure that the treatment is progressing as expected. The low-baseline LAM concentration group showed an increase in LAM concentration at week 8 of treatment. This counterintuitive increase is an important observation that warrants further investigation. One possible explanation is that samples with initially low LAM concentrations may have been suboptimal, such as being saliva-contaminated rather than true sputum. This finding suggests that in patients with low-baseline LAM levels, using sputum LAM concentrations as a TMT may be challenging.

Although Kawasaki et al. and Jones et al. conducted similar 56-day TB treatment monitoring studies [14, 16], our stratified analysis based on baseline LAM concentrations allows for a more clinically meaningful interpretation of treatment response trends, as initial levels may influence both the magnitude and pattern of LAM level trends over time. Importantly, our present study is among the first to longitudinally describe sputum LAM concentrations during TB treatment that were stratified by baseline levels, providing new insights into the potential and limitations of employing sputum LAM concentration as a biomarker for treatment monitoring.

We observed that sputum LAM concentration was significantly lower in the culture-negative group compared with the culture-positive group (10.8 pg/mL vs. 23.8 pg/mL, *P* < 0.001). This observation aligns with previous studies demonstrating a correlation between LAM concentration and culture positivity [10, 14, 16]. Jones et al. reported that sputum LAM positivity was associated with both MGIT and solid culture positivity, with an area under the curve of 0.976 and 0.979, respectively [16]. Similarly, Kawasaki et al. and Akinaga et al. showed that sputum LAM levels were inversely correlated with MGIT TTD, demonstrating their potential as an indicator of bacterial load [10, 14]. The observed gradient of sputum LAM concentration across AFB smear grades further supports its association with bacterial load. Median LAM concentration values increased progressively from smear negative samples (11.3 pg/mL) to scanty/1 + (19.7 pg/mL) and 2 +/3 + (46.7 pg/mL) samples. This trend is consistent with previous findings that demonstrated a strong correlation between sputum LAM concentration and smear grade [10, 14].

An important finding in the present study was that LAM concentrations did not significantly differ between culture-negative/AFB-negative and culture-negative/AFB-positive groups (11.1 pg/mL vs. 10.9 pg/mL, respectively; *P* = 0.4541). However, significantly higher LAM concentrations were observed in the culture-positive/AFB-positive group compared to the culture-negative/AFB-positive group (25.4 pg/mL vs. 10.9 pg/mL, respectively; *P* < 0.0001). This suggests that the presence of non-viable bacilli (as indicated by AFB positivity but culture negativity) does not substantially contribute to sputum LAM concentration. This finding is supported by Akinaga et al., who demonstrated that LAM concentration correlated strongly with culture-based assessments of bacterial load that are specific for viable MTB [10].

Although this study provides valuable insights into sputum LAM level dynamics observed during TB treatment, several limitations must be acknowledged. First, while our findings suggest the potential of LAM as a TMT alternative to AFB smear microscopy, the sample size was not adequately powered to detect differences in patient outcomes. The relatively small number of patients who experienced treatment failure limited our ability to evaluate the relationship between LAM concentrations and clinical outcomes. This limitation should be addressed in future studies. Additionally, the limited number of people living with HIV (PLWH) and drug-resistant TB cases in our cohort restricted our ability to assess any specific trends or outcomes for these subgroups of patients with TB, who generally experience poorer clinical outcomes. Additionally, we used stored samples for LAM measurements, which may have negatively affected assay performance compared to fresh samples, potentially influencing the accuracy of our findings relative to point-of-care testing [19, 20]. Sputum samples were collected as part of routine clinical care, and the quality of the samples was not ensured consistently, which could have further influenced LAM quantification. It is also possible that some follow-up samples with low or persistently low LAM concentrations reflected suboptimal sputum quality, particularly as sputum production declined during the course of treatment. In future studies, LAM measurements should ideally be performed after confirming sputum quality at each time point. In our present study, we used processed and frozen sputum samples, which limited our ability to assess their sputum quality.

## Conclusions

Our findings revealed that sputum LAM concentrations decline during TB treatment, particularly among patients with high baseline levels. Sputum LAM concentrations were associated with MGIT culture positivity and AFB smear grade and differed between culture-positive and culture-negative samples among AFB smear-positive cases. Our data highlight the potential of sputum LAM levels as a biomarker for treatment monitoring. However, the observed variability in LAM trends during TB treatment points to the complexity of LAM dynamics. Given these limitations, further prospective studies are needed to evaluate the utility of sputum LAM levels as a TMT.

## Data Availability

The datasets generated and analyzed during the current study are available from the corresponding author on reasonable request.

## Abbreviations

AFB: Acid-fast bacilli
BMI: Body mass index
CLEIA: Chemiluminescent enzyme immunoassay
ELISA: Enzyme-linked immunosorbent assay
HIV: Human immunodeficiency virus
IQR: Interquartile range
KEMRI: Kenya Medical Research Institute
LAM: Lipoarabinomannan
MGIT: Mycobacterial growth indicator tube
MTB: Mycobacterium tuberculosis
NALC: N-acetyl-L-cysteine
PLWH: People living with HIV
PTB: Pulmonary tuberculosis
RIF: Rifampin
SERU: Scientific Ethics Review Unit
TB: Tuberculosis
TMT: Treatment monitoring tool
TTD: Time to detection

## References

[1] Mulu W, Mekonnen D, Yimer M, Admassu A, Abera B. Risk factors for multidrug resistant tuberculosis patients in Amhara National Regional State. Afr Health Sci. 2015;15(2):368–77. 26124781 10.4314/ahs.v15i2.9 PMC4480497

[2] Heyckendorf J, Georghiou SB, Frahm N, Heinrich N, Kontsevaya I, Reimann M, et al. Tuberculosis treatment monitoring and outcome measures: new interest and new strategies. Clin Microbiol Rev. 2022;35(3): e0022721. 35311552 10.1128/cmr.00227-21 PMC9491169

[3] Kurbatova EV, Cegielski JP, Lienhardt C, Akksilp R, Bayona J, Becerra MC, et al. Sputum culture conversion as a prognostic marker for end-of-treatment outcome in patients with multidrug-resistant tuberculosis: a secondary analysis of data from two observational cohort studies. Lancet Respir Med. 2015;3(3):201–9. 25726085 10.1016/S2213-2600(15)00036-3 PMC4401426

[4] Horne DJ, Royce SE, Gooze L, Narita M, Hopewell PC, Nahid P, et al. Sputum monitoring during tuberculosis treatment for predicting outcome: systematic review and meta-analysis. Lancet Infect Dis. 2010;10(6):387–94. 20510279 10.1016/S1473-3099(10)70071-2 PMC3046810

[5] Nema V. Tuberculosis diagnostics: challenges and opportunities. Lung India. 2012;29(3):259–66. 22919166 10.4103/0970-2113.99112 PMC3424866

[6] MacLean ELH, Zimmer AJ, den Boon S, Gupta-Wright A, Cirillo DM, Cobelens F, et al. Tuberculosis treatment monitoring tests during routine practice: study design guidance. Clin Microbiol Infect. 2024;30(4):481–8. 38182047 10.1016/j.cmi.2023.12.027

[7] Zimmer AJ, Lainati F, Aguilera Vasquez N, Chedid C, McGrath S, Benedetti A, et al. Biomarkers that correlate with active pulmonary tuberculosis treatment response: a systematic review and meta-analysis. J Clin Microbiol. 2022;60(2): e0185921. 34911364 10.1128/jcm.01859-21 PMC8849205

[8] Broger T, Koeppel L, Huerga H, Miller P, Gupta-Wright A, Blanc FX, et al. Diagnostic yield of urine lipoarabinomannan and sputum tuberculosis tests in people living with HIV: a systematic review and meta-analysis of individual participant data. Lancet Glob Health. 2023;11(6):e903–16. 37202025 10.1016/S2214-109X(23)00135-3

[9] Dheda K, Davids V, Lenders L, Roberts T, Meldau R, Ling D, et al. Clinical utility of a commercial LAM-ELISA assay for TB diagnosis in HIV-infected patients using urine and sputum samples. PLoS ONE. 2010;5(3): e9848. 20352098 10.1371/journal.pone.0009848 PMC2844421

[10] Akinaga A, Takahashi M, Yamazaki T, Chikamatsu K, Matsushita S, Hashimoto Y, et al. Development and preliminary evaluation toward a new tuberculosis treatment monitoring tool: the PATHFAST TB LAM Ag assay. J Clin Microbiol. 2024;62(8): e0062924. 39028178 10.1128/jcm.00629-24 PMC11323533

[11] Pereira Arias-Bouda LM, Nguyen LN, Ho LM, Kuijper S, Jansen HM, Kolk AH. Development of antigen detection assay for diagnosis of tuberculosis using sputum samples. J Clin Microbiol. 2000;38(6):2278–83. 10834989 10.1128/jcm.38.6.2278-2283.2000 PMC86781

[12] Chan ED, Reves R, Belisle JT, Brennan PJ, Hahn WE. Diagnosis of tuberculosis by a visually detectable immunoassay for lipoarabinomannan. Am J Respir Crit Care Med. 2000;161(5):1713–9. 10806179 10.1164/ajrccm.161.5.9908125

[13] Peter JG, Cashmore TJ, Meldau R, Theron G, van Zyl-Smit R, Dheda K. Diagnostic accuracy of induced sputum LAM ELISA for tuberculosis diagnosis in sputum-scarce patients. Int J Tuberc Lung Dis. 2012;16(8):1108–12. 22710609 10.5588/ijtld.11.0614 PMC5463742

[14] Kawasaki M, Echiverri C, Raymond L, Cadena E, Reside E, Gler MT, et al. Lipoarabinomannan in sputum to detect bacterial load and treatment response in patients with pulmonary tuberculosis: analytic validation and evaluation in two cohorts. PLoS Med. 2019;16(4): e1002780. 30978194 10.1371/journal.pmed.1002780 PMC6461223

[15] Cho SN, Shin JS, Kim JD, Chong Y. Production of monoclonal antibodies to lipoarabinomannan-B and use in the detection of mycobacterial antigens in sputum. Yonsei Med J. 1990;31(4):333–8. 2127645 10.3349/ymj.1990.31.4.333

[16] Jones A, Saini J, Kriel B, Via LE, Cai Y, Allies D, et al. Sputum lipoarabinomannan (LAM) as a biomarker to determine sputum mycobacterial load: exploratory and model-based analyses of integrated data from four cohorts. BMC Infect Dis. 2022;22(1):327. 35366820 10.1186/s12879-022-07308-3 PMC8976459

[17] World Health Organization. Laboratory Quality Stepwise Implementation tool [Internet]. [cited 2025 Jun 9]. Available from: https://extranet.who.int/lqsi/content/tb-sop-specimen-processing-culture.

[18] Akinaga A, Diacon AH, Ocloo R, Yanagida A, Yoda N, Kawasaki M, et al. Quantification of bactericidal activity using the PATHFAST TB LAM Ag assay during the first 14 days of pulmonary tuberculosis treatment. Front Antibiot. 2025;4:1574688. 40443956 10.3389/frabi.2025.1574688 PMC12120838

[19] Broger T, Muyoyeta M, Kerkhoff AD, Denkinger CM, Moreau E. Tuberculosis test results using fresh versus biobanked urine samples with FujiLAM. Lancet Infect Dis. 2020;20(1):22–3. 31876492 10.1016/S1473-3099(19)30684-X

[20] Connelly JT, Grant B, Munsamy V, Pym A, Somoskovi A. Lipoarabinomannan point-of-care tests: evaluation with fresh samples needed. Lancet Infect Dis. 2019;19(10):1053. 31559954 10.1016/S1473-3099(19)30475-X

